# Thumb Sucking

**Published:** 1890-02

**Authors:** 


Thumb Sucking.—Mr. S. O. Eades (*British Med. Journal*) disputes the notion that thumb sucking is injurious. He says: "I always advise parents to encourage this habit, especially during dentition. Sucking the thumb causes the salivary glands to pour out their secretions, thus moistening the mouth and aiding digestion; the pressure of the thumb eases, while the teeth are "bleeding," the irritation and pain of the gums, and helps, when the teeth are sufficiently advanced, to bring them through. Sucking of the thumb, moreover, makes a cross infant contented and happy, and frequently induces a restless babe to fall into a sweet, refreshing sleep. After dentition is completed it is likely to become a habit with a child; in that case it may be readily cured by smearing its thumb with a paste of aloes and water; one or two applications will suffice, as after tasting the bitter it will eschew its former enjoyment. I may add that thumb sucking, in my opinion, is far preferable to ivory, india rubber rings, nipples, etc., we see so frequently given to these poor mortals by their loving mothers."

